# Serum exosomal circCCDC66 as a potential diagnostic and prognostic biomarker for pituitary adenomas

**DOI:** 10.3389/fonc.2023.1268778

**Published:** 2023-11-30

**Authors:** Xiao Yue, Fengming Lan, Weiping Liu

**Affiliations:** ^1^ Department of Neurosurgery, Xijing Hospital, Air Force Medical University, Xi’an, Shaanxi, China; ^2^ National Cancer Center/National Clinical Research Center for Cancer/Cancer Hospital and Shenzhen Hospital, Chinese Academy of Medical Sciences and Peking Union Medical College, Shenzhen, China

**Keywords:** pituitary adenoma, biomarker, circRNA, circCCDC66, serum

## Abstract

**Purpose:**

Circular RNAs (circRNAs) play an important role in tumorigenesis, and exosomal circRNAs have the potential to be novel biomarkers for cancer diagnosis. Here, we are committed to reveal serum exosomal circCCDC66 as a noninvasive biomarker to diagnose and predict recurrence in pituitary adenoma (PA).

**Methods:**

A total of 90 PA patients and 50 healthy subjects were enrolled for clinical validation. Exosomes were extracted from the serum and validated by transmission electron microscopy, nanoparticle tracking analysis, and Western blot assay. The expression of circCCDC66 in serum exosomes was assessed using quantitative real-time PCR (qRT-PCR), and correlations between circCCDC66 expression and clinicopathological factors were analyzed. The reliability of serum exosomal circCCDC66 to diagnose PA was evaluated using receiver operating characteristic (ROC) analysis.

**Results:**

Initially, an obviously significantly increasing level of serum exosomal circCCDC66 was verified in the PA specimens compared with healthy controls. Importantly, serum exosomal circCCDC66, which was secreted and released by PA cells, could monitor tumor dynamics and serve as a potentially prognostic biomarker during treatment. Additionally, ROC curve analysis was performed and the corresponding area under the curve (AUC) values were used to confirm the ability of serum exosomal circCCDC66 as a potentially diagnostic and prognostic biomarker for PA patients. Importantly, the progression-free survival was much longer in the low serum exosomal circCCDC66 group than in the high serum exosomal circCCDC66 group.

**Conclusion:**

Serum exosomal circCCDC66 is abnormally elevated and may serve as a promising factor for the diagnosis of and predicting prognosis in PA patients.

## Introduction

Following glioma, pituitary adenomas (PAs) have the second-highest incidence rate among primary intracranial tumors, accounting for 10%–25% in central nervous system neoplasms (1). Though PAs are classified as benign tumors, the majority of symptoms including hypopituitarism, hypersecretion of hypophyseal hormone, and visual and neurological deficits have a significant impact on patients. PA could be divided into nonfunctional and functional disease according to secreting hormone or not (2). As reported, there are approximately 40% of PA patients exhibiting invasive behaviors and resulting in poor prognosis. Options for treatment of PA include maximal surgical resection, radiation therapy, and systemic therapies based on cancer type, special stage, and any other situations. Early and accurate diagnosis can reduce the morbidity and mortality of PA. Recent advances in inherited genetic vulnerability and specifically biological features have broadened the understanding of pituitary tumorigenesis (3). However, the specific molecular pathogenesis and targeted treatment options are still unclear in PA. Consequently, an insightful understanding of molecular levels and potential targets in PA is largely unknown and deserves more exploration (4).

Exosomes, which are one of extracellular vehicles (EVs) with a diameter range of 30–160 nm, are reported recently as the essentially diagnostic factors in a variety of diseases, including benign and malignant tumors, respiratory disorders, neurological disease, and organ-specific autoimmune disease (5). Exosomes widely exist in common body fluids including blood serum, urine, saliva, and peritoneal and cerebrospinal fluid. Exosomes could be secreted by almost all somatic cells from the endosomal pathway experiencing the procedure of endocytosis, merging, and releasing (6). More importantly, a large number of studies have shown that exosomes are stable and excellent biomarkers to monitor and predict many diseases due to containing a variety of disease-specific proteins, DNAs, miRNAs, lncRNAs, and circRNAs (7). At present, studies have confirmed that circRNAs encased in exosomes are more resistant to the degradation of RNase R and thus have longer half-times due to the specially closed loop structure, which indicates that circRNAs could be outstanding signaling molecules for cell–cell communication, and also significantly diagnostic biomarkers and potentially prognostic targets for all kinds of disease (8).

CircRNA, which is a unique particular endogenous RNA with a covalently closed loop, is generated from the back-splicing mechanism of pre-mRNAs. CircRNA is considered as a functional molecule in tumor biology and functional regulation of diverse gene expression (9). A number of studies have demonstrated that circulating exosomal circRNA are an essential factor in the biological and pathophysiological cancer process and the changing tumor microenvironment (10). In the present study, we are aimed to investigate serum exosomal circCCDC66 as a potentially diagnostic and prognostic biomarker in PA patients.

## Materials and methods

### Patients and clinical samples

A total of 90 cases of primary PA patients who received surgical resection and 50 cases of healthy volunteers were enrolled at the First Affiliated Hospital (Xijing Hospital) of the Air Force Medical University between June 2006 and May 2010. The inclusion criteria were as follows (1): the primary tumor (2); both radiological and pathological diagnosis verified as PA (3); no surgical or other special treatment; and (4) with complete clinicopathological information. The following were the exclusion criteria (1): together with severe liver, kidney, heart, and lung insufficiency (2); combined with other cancer; and (3) lost to follow-up. All patients were followed up by radiographical and clinical examinations till July 2022, and the median follow-up time is 101.6 months. The follow-up interval began on the date of the patient’s first tumor resection and ended on the date of tumor recurrence or last follow-up. Tumor recurrence was confirmed by clinical and imaging findings or histology analysis of specimens from the second surgery. Furthermore, another 15 normal human anterior pituitary glands were acquired from the donation process as the normal group. This study was approved by the ethics committee of First Affiliated Hospital (Xijing Hospital) belonging to the Air Force Medical University (No. 81627806). Informed consent was acquired from all patients, and the study was performed in accordance with the principles of the Declaration of Helsinki.

Serum samples were obtained from each patient before and on the 7th day after surgery. The changes in exosomal circCCDC66 between post-operation and recurrence from 30 recurrent patients were monitored. For serum specimens, peripheral venous blood (5 mL each) was stored at 4°C within 1 h after collection and centrifuged at 3000 g for 10 min at 4°C. Next, the supernatant was further centrifuged at 12,000 *g* for 10 min at 4°C to remove cell debris. Lastly, the supernatant fluids were transferred to RNase-free centrifuge tubes, and stored at −80°C for further analyses.

All tissue specimens were carried out and made anonymous by complying with the ethical and legal standards. The collected clinical specimens were first instantly frozen in liquid nitrogen and then stored in a −80°C refrigerator before examination. Frozen tissue specimens of PA were subjected to RNA examining after operation.

### Exosome isolation

Enrichment and purification of exosomes from serum were conducted using the ExoQuick™ ULTRA EV Isolation Kit (cat: EQULTRA-20A-1, System Biosciences, Mountain View, CA, USA) according to the manufacturer’s instructions. All exosomes were stored at −80°C.

### Exosome identification

Transmission electron microscopy (TEM) and nanoparticle tracking analysis (NTA) were used to observe the morphological characteristics of the purified exosomes as described previously (11).

Expression of exosomal markers such as protein CD63 and TSG101 was analyzed by Western blotting. RIPA lysis buffer (cat: 89901, Thermo Scientific) was used to extract total proteins. Proteins were separated using 10% sodium dodecyl sulfate polyacrylamide (SDS-PAGE) gel electrophoresis, followed by transferring onto the polyvinylidene difluoride (PVDF) membranes (Millipore, Billerica, MA, USA). Following blockage using 5% skimmed milk for 1 h, the PVDF membranes were incubated overnight with primary antibodies at 4°C. Finally, the combined signals were detected by Chemistar™ High-sig ECL Western Blot Substrate (cat: P10200, Tanon, Shanghai, China) after incubation of corresponding HPR-labeled secondary antibody for 2 h at room temperature. The antibodies CD63 (1:2,000, ab134045), TSG101 (1:2,000, ab30871), and HRP-conjugated IgG anti-rabbit (1:6,000, ab205718) were commercially acquired from Abcam (Cambridge, MA, USA).

### RNA extraction and quantitative real-time PCR

The total RNA from tissue specimens was extracted using the TRIzol (Invitrogen, Carlsbad CA, USA) method. Exosomal RNA was isolated from serum utilizing the exoRNeasy Serum/Plasma Maxi Kit (Qiagen, Germany) following the manufacturer’s protocol. Next, the purity and concentration of RNA samples were evaluated using a NanoDrop 2000 spectrophotometer (Thermo Scientific, Wilmington, DE, USA), and the OD260/OD280 ratio that ranged from 1.8 to 2.0 indicated high purity. Total RNA was reverse transcribed to synthesize cDNA with random primers using a Prime Script RT Reagent Kit (TaKaRa, Dalian, China). Thereafter, the qPCR was executed by using Master Mix SYBR Green RT-PCR Super Mix (Tiangen Biotech Co., Ltd.) on the ABI 7500 Real-time PCR system (Applied Biosystems, CA, USA). The Glyceraldehyde-3-phosphate dehydrogenase (GAPDH) gene was used as an internal reference of mRNAs. PCR primers are listed as follows: circCCDC66 F: 5′-ACC TAC AAC CGG AAG CCA G-3′, R: 5′-AGC AGT ACT GTT TCC TGA TGC-3′. GAPDH F: 5′-GGA GCG AGA TCC CTC CAA AAT-3′, R: 5′-GGC TGT TGT CAT ACT TCT CAT GG-3′. The relative expression levels of circCCDC66 were calculated using the 2^−ΔΔCt^ method. All experiments were repeated three times.

### Statistical analysis

Statistical Program for Social Sciences (SPSS) 22.0 software (SPSS, Chicago, IL, USA) and GraphPad Prism 8.0 Software (GraphPad Software, La Jolla, CA, USA) were used for all of the statistical analyses. The Student’s *t*-test or one-way ANOVA was used to analyze qRT‐PCR results between the PA and control groups. The chi-square or Fisher’s exact test was used to analyze the correlations between exosomal circCCDC66 expression and the clinicopathological factors of PA patients. The correlation of circCCDC66 expression levels in PA tissues and serum samples was performed by Spearman correlation coefficient. The receiver operating characteristic (ROC) curve and the Kaplan–Meier survival plot were established to evaluate the diagnostic and prognostic capacity of exosomal circCCDC66. Statistical significance was determined as *p* < 0.05 (*) or *p* < 0.01 (**).

## Results

### Extraction and characterization of serum exosomes

**Figure 1:**
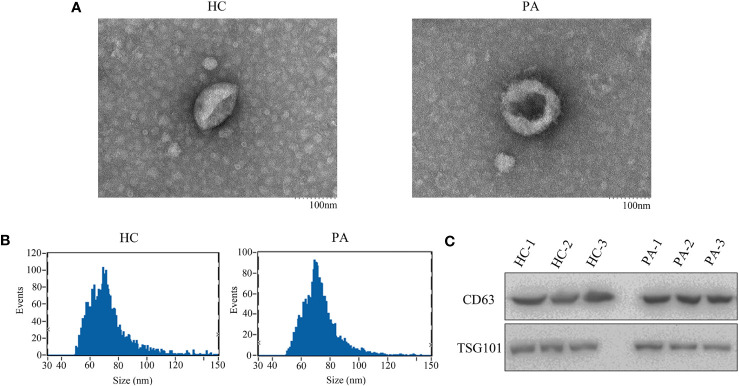
The shapes of serum exosomes from healthy controls and pituitary adenoma (PA) patients **(A)**. **(B)** Size analyses of serum exosomes from healthy controls and PA patients were performed by nanoparticle tracking analysis. **(C)** The exosomal markers CD63 and TSG101 were measured by Western blot.

Serum exosomes were extracted from PA patients and healthy control group samples to explore the potentially diagnostic biomarkers of exosomal circRNA in PA. As shown in **Figure 1A**, the exosomes characterized as cup-shaped morphology were observed with an atypical size of 30–160 nm. The diameters of exosomes in the healthy controls and PA patients were 73.02 and 74.12 nm, respectively (**Figure 1B**). Western blotting was applied to detect the expression of exosomal markers CD63 and TSG101, and the results proved that the two markers were all highly enriched in the extracted exosomes whether from healthy controls or PA patients (**Figure 1C**). Taken together, the above results, which confirmed the validity and purity of exosome extraction from serum in this study, served as a good foundation for further exploration.

### CircCCDC66 expression was increased in the serum exosome and specimens of PA patients

**Figure 2:**
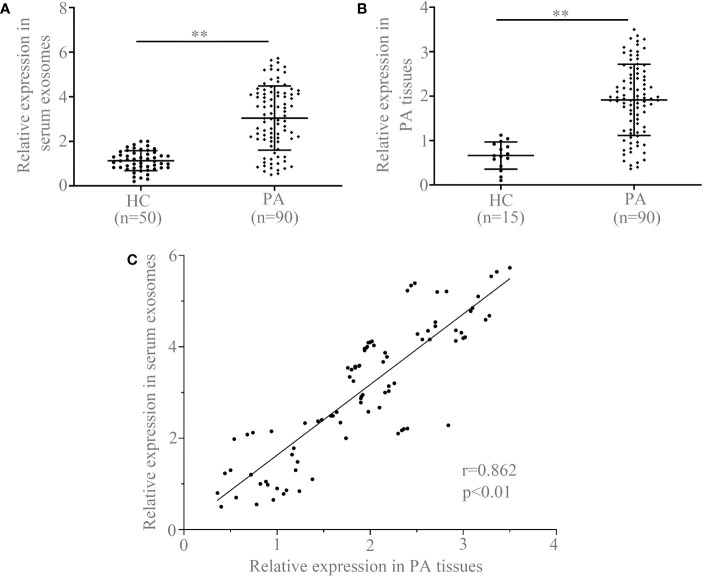
Serum exosomal circCCDC66 **(A)** and tissue circCCDC66 expression **(B)** in PA patients and healthy controls analyzed by qRT-PCR. **(C)** The relationship of circCCDC66 expression levels in PA tissues and serum samples (*r* = 0.862). **p < 0.01.

In order to evaluate the diagnostic value and prognostic significance of circCCDC66 in PA, quantitative real-time PCR (qRT-PCR) was initially applied to examine the expression of circCCDC66 in serum exosomes. Levels of serum exosomal circCCDC66 were examined in **Figure 2A** to compare the difference between PA patients (*n* = 90) and healthy controls (*n* = 50). Analysis of the expression of exosomal circCCDC66 revealed an obviously significant upregulation in PA patients in contrast to healthy controls after normalization (*p* < 0.01, **Figure 2A**). The expression of circCCDC66 in PA tissue samples was also measured, as shown by the results in serum exosome, and circCCDC66 was significantly upregulated in PA specimens (**Figure 2B**). Pearson’s correlation coefficient analysis revealed a significant positive correlation between the expression of PA tissues and serum samples (*r* = 0.864, *p* < 0.01, **Figure 2C**). CircCCDC66 expression was totally increased in the serum exosome and tissue specimens of PA patients.

### Serum exosomal circCCDC66 expressions monitored tumor dynamics and served as a potentially prognostic biomarker for PA patients

**Figure 3:**
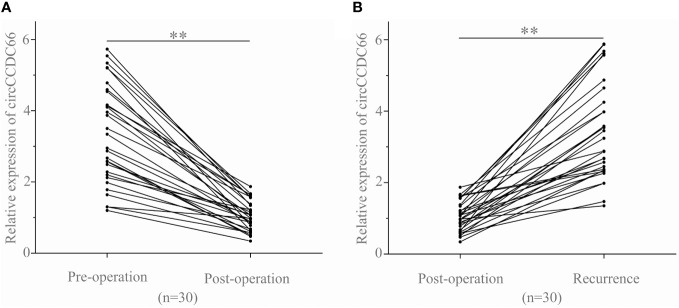
Serum exosomal circCCDC66 levels reflect tumor dynamics. **(A)** Serum exosomal circCCDC66 expression levels before and after surgery. **(B)** Comparison of serum exosomal circCCDC66 levels in samples obtained post-operatively and after recurrence. **p < 0.01.

To further investigate whether serum exosomal circCCDC66 expressions are associated with tumor dynamics, we analyzed serum exosomal circCCDC66 expressions in 30 recurrent patients at different time periods. As observed, serum exosomal circCCDC66 expressions were statistically downregulated following resection of the primary tumor (*p* < 0.01, **Figure 3A**). However, the expression levels of exosomal circCCDC66 were remarkably increased at the time of PA recurrence (*p* < 0.01, **Figure 3B**). Collectively, serum exosomal circCCDC66, which was secreted and released by PA cells, could monitor tumor dynamics and serve as a potentially prognostic biomarker during treatment.

### Diagnostic performance of serum exosomal circCCDC66 in PA patients

**Figure 4:**
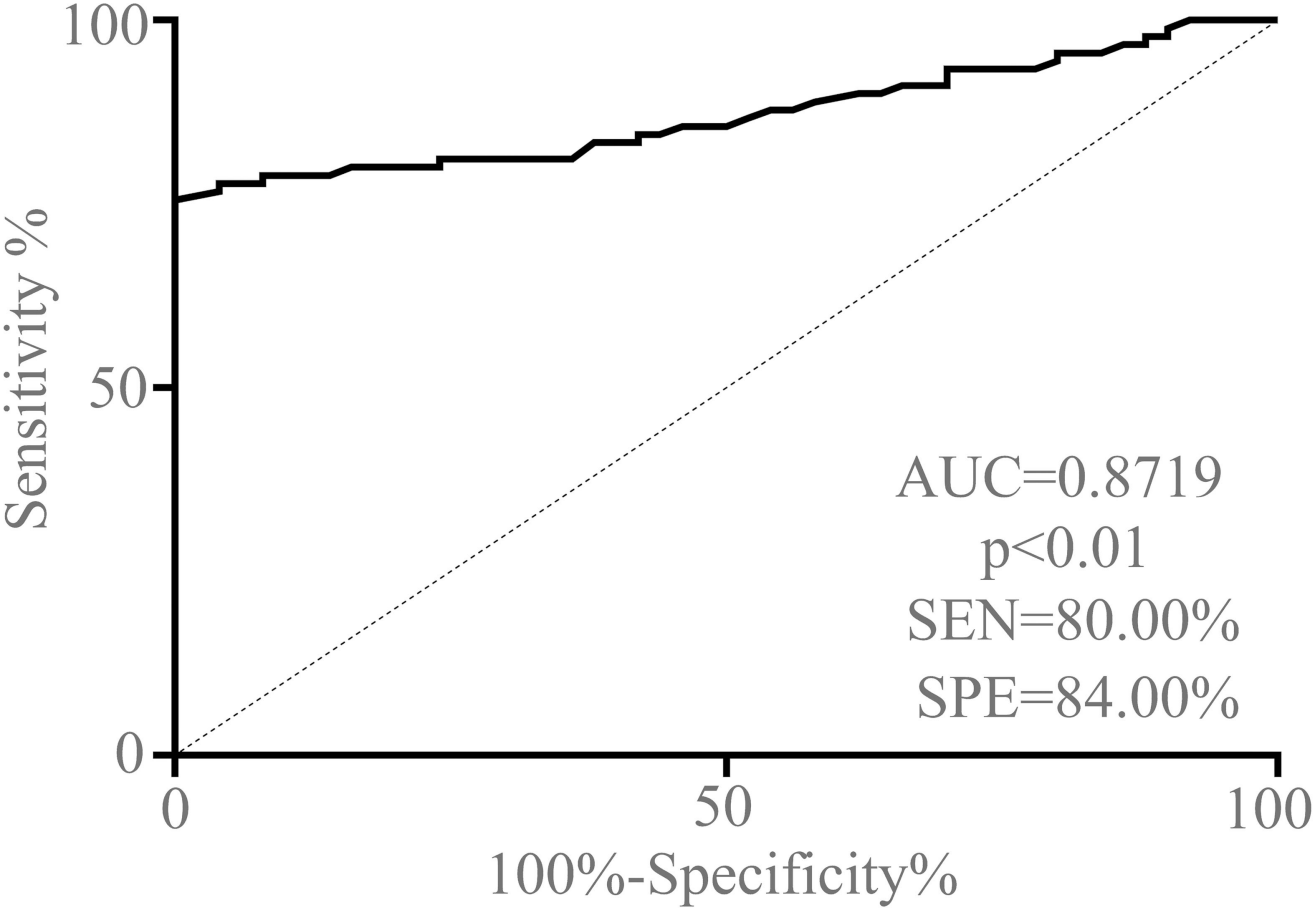
ROC curve analysis based on serum exosomal circCCDC66 levels for distinguishing PA patients from normal controls.

The receiver operating characteristic (ROC) curve analysis and the corresponding area under the curve (AUC) values were performed to confirm the ability of serum exosomal circCCDC66 as a potentially diagnostic and prognostic biomarker for PA patients. As demonstrated in **Figure 4**, the AUC value of serum exosomal circCCDC66 levels was 0.8719 (95% CI: 0.8134–0.9304), which suggested that exosomal circCCDC66 could discriminate PA patients from healthy controls (*p* < 0.01). At the optimal cutoff value, the sensitivity, specificity, and positive and negative predictive values to distinguish PA patients were 80.00%, 84.00%, 86.64%, and 79.53%, respectively. These findings indicated that serum exosomal circCCDC66 could be a promising diagnostic biomarker to effectively monitor the PA occurrence.

### Association of serum exosomal circCCDC66 expression with the prognosis of PA patients

**Figure 5:**
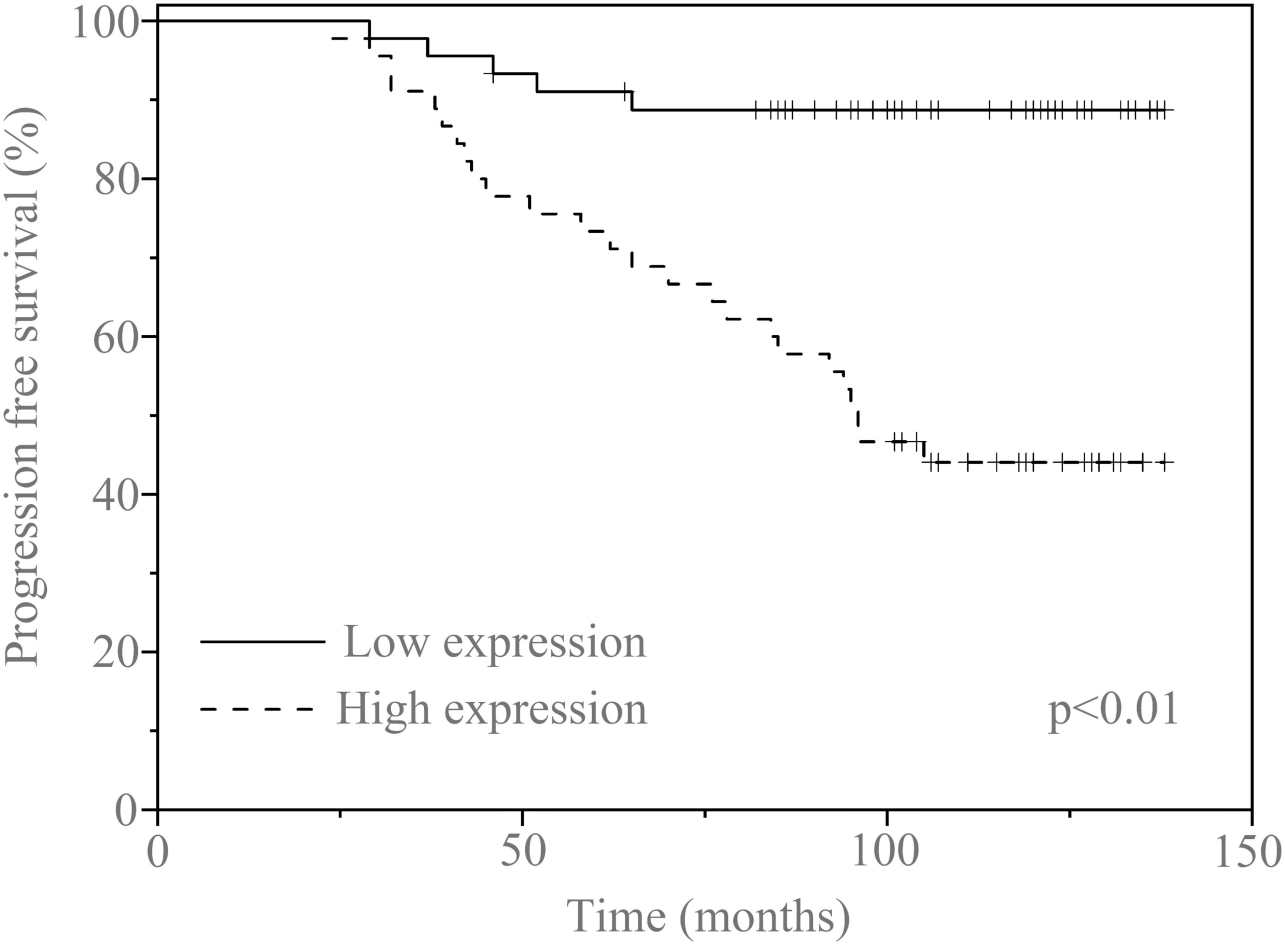
Kaplan–Meier survival curves for patients with PA and high (*n* = 45) or low (*n* = 45) serum exosomal circCCDC66 expression levels.

In this cohort, we were engaged to confirm the prognostic function of serum exosomal circCCDC66 in PA patients. The clinical characteristics of the recruited PA patients are summarized in **Table 1** according to the median expression of serum exosomal circCCDC66. The follow-up information of the patients was updated to July 2022. There were no statistically significant differences in age and gender distribution, while Knosp classification, tumor size, and recurrence were associated with the expression of serum exosomal circCCDC66 in PA (*p* < 0.01). Interestingly, the progression-free survival (PFS) was much longer in the low serum exosomal circCCDC66 group compared with the high serum exosomal circCCDC66 group (*p* < 0.01, **Figure 5**). Totally, these results provided lines of evidence that serum exosomal circCCDC66 may serve as a potential factor for prognosis prediction.

**Table 1:** The correlation of serum exosomal circCCDC66 expression levels with clinicopathological factors.

Characteristic	Total no.	circCCDC66 expression		
		Low (*n*)	High (*n*)	*p*
Total no.	90	45	45	
Age (years)^+^				
≤45	46	22	24	NS
>45	44	23	21	
Gender				
Male	43	20	23	NS
Female	47	25	22	
Types				
PRL-PA	28	13	15	NS
ACTH-PA	10	6	4	
GH-PA	22	10	12	
NFPA	30	16	14	
Knosp				
0–II	46	37	9	<0.01
III–IV	44	8	36	
Tumor size (cm)				
<1 cm	24	23	1	<0.01
1–3 cm	34	20	14	
>3 cm	32	2	30	
Recurrence				
Yes	30	5	25	<0.01
No	60	40	20	

^+^Median age was 45 years.

PRL-PA, prolactin pituitary adenoma; ACTH-PA, adrenocorticotrophic hormone pituitary adenoma; GH-PA, growth hormone pituitary adenoma; NFPA, nonfunctioning pituitary adenoma.

## Discussion

Serum biomarkers could supply a precise evaluation to diagnose and predict the various tumors for survival improvements (12). With an improvement in technology of liquid biopsy, a wide variety of public literature has confirmed that tumor-specific proteins and RNAs that are encapsulated in exosomes exhibit enormous potentially valuable and promising predicable abilities in the diagnosis and prognosis judgment of a great many diseases. Importantly, as a circulating tumor biomarker, exosomes are more stable because of a lipid bilayer membrane that can prevent the degradation of internal molecules from the external environment (13). As for now, numerous high-risk genes were reported as the diagnostic and prognostic factor in PA. Serum anterior gradient-2 can be used as a potential biomarker screening in the diagnosis of PA (14). The single-cell transcriptome and single-cell multi-omics analyses revealed that novel tumor-related genes, such as AMIGO2, ZFP36, BTG1, and DLG5, were mainly upregulated in pituitary neuroendocrine tumors (15). Exo-MMP1 correlated with the characteristic invasiveness of non-functional PA and may be a novel therapeutic target (16).

To date, substantial studies reported that exosome is a non-invasive, sensitive, and specific biomarker in the tumor’s occurrence and development. has-miR-619-5p ahashsa-miR-4454 in plasma-derived exosomes have the potential to be used as biomarkers for the early diagnosis of lung adenocarcinoma (17). Exosomal circ_0009910 regulates proliferation, cell cycle, and apoptosis of acute myeloid leukemia cells by interfering with the miR-5195-3p/GRB10 axis (18). Exosome miR-552 promotes laryngocarcinoma cells’ malignant progression by the regulation of the PTEN/TOB1 axis (19). Liver fibrosis-derived exosomal miR-106a-5p facilitates the malignancy by targeting SAMD12 and CADM2 in hepatocellular carcinoma (20). Tumor exosome-derived lncRNA HOTAIR can be used as a potential biomarker for the diagnosis and treatment of gastric cancer (21). The serum-derived exosomal PD-L1 levels were higher in metastatic pancreatic cancer than locally advanced disease. Higher serum exosomal PD-L1 levels in advanced pancreatic cancer patients suggested worse survival outcomes and may have clinical implications (22).

Accumulating lines of evidence demonstrate that circRNAs are widely distributed in the cytoplasm and nucleus and function as effective treatment options in all kinds of disease (23). CircRNAs, which are typically expressed in specific tissues, have been proposed as potential prognostic or diagnostic biomarkers for various cancers. Specifically, the circular RNA F-circEA derived from the EML4-ALK fusion gene has emerged as a novel liquid biopsy biomarker for non-small cell lung cancer (24). Genome-wide CRISPR screen identifies HNRNPL as a prostate cancer dependency regulating RNA splicing (25). Epigenetic silencing of CDR1as drives IGF2BP3-mediated melanoma invasion and metastasis (26). N6-methyladenosine modification of circNSUN2 facilitates cytoplasmic export and stabilizes HMGA2 to promote colorectal liver metastasis (27). Spatial expression analyses of the putative oncogene ciRS-7 in cancer reshape the miRNA sponge theory (28). Circular RNA profiling reveals an abundant circHIPK3 that regulates cell growth by sponging multiple miRNAs (29). circNDUFB2 inhibits non-small cell lung cancer progression via destabilizing IGF2BPs and activating anti-tumor immunity (30). A novel protein encoded by circMAPK1 inhibits progression of gastric cancer by suppressing activation of MAPK signaling (31).

CircCCDC66, also named circ_0001313, is a novel oncogenic circRNA due to the enrichment in a variety of tumors. The current studies about circCCDC66 mainly focus on the expression, biological functions, and regulatory mechanisms in the diagnosis prediction, prognosis assessment, and molecular treatment of various cancers (32). As reported, circCCDC66 promotes thyroid cancer cell proliferation, migratory and invasive abilities, and glycolysis through the miR-211-5p/PDK4 axis (33). Hypoxia‐induced circCCDC66 promotes the tumorigenesis of colorectal cancer via the miR‐3140/autophagy pathway (34). CircCCDC66 promotes glioma proliferation by acting as a ceRNA for miR-320a to regulate FOXM1 expression (35). STAT3-induced upregulation of circCCDC66 facilitates the progression of NSCLC by targeting the miR-33a-5p/KPNA4 axis (36). CircCCDC66 contributes to the malignant phenotype of osteosarcoma by sponging miR-338-3p to upregulate the expression of PTP1B (37). CircCCDC66 regulates osteoarthritis progression by targeting miR-3622b-5p (38). CircCCDC66 is related to poor prognosis and promotes the growth and metastasis of colon cancer (39).

Here, we reveal exosomal circCCDC66 as a noninvasive biomarker to diagnose and predict recurrence in PA. Initially, an obviously significantly increasing level of serum exosomal circCCDC66 was verified in the PA specimens compared with healthy controls. Importantly, serum exosomal circCCDC66, which was secreted and released by PA cells, could monitor tumor dynamics and serve as a potentially prognostic biomarker during treatment. Additionally, ROC curve analysis was performed and the corresponding AUC values were used to confirm the ability of serum exosomal circCCDC66 as a potentially diagnostic and prognostic biomarker for PA patients. Importantly, the PFS was much longer in the low serum exosomal circCCDC66 group than in the high serum exosomal circCCDC66 group.

In summary, our study indicates that exosomal circCCDC66 is highly expressed in PA patients and might be a promising noninvasive biomarker for the diagnostic and prognostic evaluation of PA.

## Data availability statement

The raw data supporting the conclusions of this article will be made available by the authors, without undue reservation.

## Ethics statement

The studies involving humans were approved by the Ethics Committee of the Air Force Medical University. The studies were conducted in accordance with the local legislation and institutional requirements. Written informed consent for participation in this study was provided by the participants’ legal guardians/next of kin. Informed consent was obtained from all individual participants included in the study.

## Author contributions

XY: Conceptualization, Data curation, Formal Analysis, Investigation, Writing – original draft. FL: Data curation, Formal Analysis, Investigation, Writing – original draft. WL: Conceptualization, Funding acquisition, Methodology, Project administration, Resources, Supervision, Visualization, Writing – original draft.
